# Mental and somatic health burdens of hypochondriacal disorder in higher education: national study among Norwegian students

**DOI:** 10.1192/bjo.2025.68

**Published:** 2025-05-15

**Authors:** Kari-Elise Frøystad Veddegjærde, Jens Christoffer Skogen, Ingvard Wilhelmsen, Børge Sivertsen

**Affiliations:** Department of Clinical Science, University of Bergen, Bergen, Norway; Department of Psychiatry and Drug Abuse, Ålesund Hospital, Møre og Romsdal Health Trust, Ålesund, Norway; Department of Health Promotion, Norwegian Institute of Public Health, Bergen, Norway; Center for Alcohol & Drug Research, Stavanger University Hospital, Stavanger, Norway; Centre for Evaluation of Public Health Measures, Norwegian Institute of Public Health, Oslo, Norway; Department of Research & Innovation, Helse-Fonna HF, Haugesund, Norway

**Keywords:** Hypochondriacal disorder, university students, depression, mental health, somatic symptoms

## Abstract

**Background:**

Hypochondriacal disorder involves persistent anxiety about suffering from an undetected serious medical condition, despite medical reassurance. Hypochondriacal disorder significantly affects social relationships, occupational functioning and personal well-being. In university settings, where mental health concerns are prevalent, insights into prevalence of hypochondriacal disorder and associations with depression and other health challenges are essential.

**Aims:**

This study examines the prevalence and correlates of hypochondriacal disorder among Norwegian university students, focusing on its associations with depression, mental distress and somatic symptom burden.

**Method:**

The 2022 Students’ Health and Wellbeing Study, a national survey of Norwegian higher education students, included 59 536 participants aged 18–35. Participants were categorised based on a pre-defined diagnostic list of mental and somatic concerns, and participants were grouped as follows: hypochondriacal disorder only, depression only, comorbid hypochondriacal disorder and depression and controls. Validated instruments included the Somatic Symptom Scale-8, the Hopkins Symptoms Checklist, the Satisfaction With Life Scale, an abbreviated version of the University of California, Los Angeles, Three-Item Loneliness Scale and four items on suicidal ideation.

**Results:**

Hypochondriacal disorder was reported by 0.86% (*n* = 457) of participants, with 52% also reporting depression. Those with hypochondriacal disorder had significantly worse mental and somatic health outcomes, especially when comorbid with depression, including elevated distress, suicidality, insomnia and poor quality of life.

**Conclusion:**

Although uncommon, hypochondriacal disorder is linked to severe mental and somatic health burdens, particularly when co-occurring with depression. These findings highlight the need for integrated mental health strategies in academic settings to address hypochondriacal disorder and its frequent comorbidities.

Hypochondriacal disorder is characterised by a continuous and intense worry about having a severe medical condition that has gone undiagnosed, even when medical evaluations provide reassurance.^1^ This condition often involves frequent monitoring of bodily symptoms, misinterpretation of these symptoms and safety-seeking behaviours.^1–3^

The terminology and conceptualisation of hypochondriacal disorder and health anxiety remain inconsistent across the literature. Some researchers use these terms synonymously, while others emphasise distinctions based on cognitive mechanisms or the prominence of fear versus conviction.^3^ This study defines ‘hypochondriacal disorder’ as a self-reported, persistent preoccupation with serious illness, ensuring clarity while acknowledging potential misalignment with other conceptual frameworks.

Prevalence estimates for hypochondriacal disorder vary widely, reflecting differences in diagnostic criteria and study design. Lifetime prevalence rates have been reported to reach up to 5.7%,^4^ while current prevalence has been reported as high as 3.4%.^2,5,6^ Evidence suggests that the disorder disproportionately affects individuals in primary care settings, where it drives a substantial share of healthcare utilisation. A meta-analysis of over 22 000 university students found a significant increase in health anxiety levels between 1985 and 2017, with the estimated prevalence of clinically significant health anxiety rising from 9% to 15% during this period,^7^ highlighting the need for focused research in this demographic.

University students undergo significant developmental changes and heightened stress, which may increase their vulnerability to health-related anxieties. Hypochondriacal disorder often co-occurs with other mental health issues, particularly depression, creating a complex interplay between mental health and somatic concerns.^8^ Somatic concerns – such as gastrointestinal, cardiopulmonary and neurological symptoms – are common and often intertwined with health-related anxiety. The disorder’s isolating nature, compounded by loneliness and reduced quality of life, further underscores its clinical and debilitating impact.^9^ Notably, this association extends to an elevated risk of suicidality, as demonstrated in a recent Swedish study reporting a fourfold increased risk of suicide among individuals with hypochondriacal disorder.^10^

This study utilises data from the SHoT (Students’ Health and Wellbeing Study), a national survey of Norwegian university students, to examine the prevalence and correlates of hypochondriacal disorder, with a focus on its comorbidity with depression. Participants were categorised into four groups: (a) hypochondriacal disorder only, (b) depression only, (c) comorbid hypochondriacal disorder and depression and (d) controls with neither condition. By analysing mental distress, somatic symptoms, suicidality, insomnia, quality of life and loneliness across these groups, this research leverages the overall scope of SHoT data to inform targeted interventions for improving student well-being.

## Method

### Study design, participants and setting

The SHoT is a nationwide survey examining student health in Norwegian higher education, established and financed by Norway’s three largest student welfare organisations: SiO (Student Welfare Organization of Oslo and Akershus), Sammen (Student Welfare Organization of Western Norway) and SiT (Student Welfare Organization of Trondheim). These organisations provide essential services, including housing, counselling and healthcare, to support student well-being. While these organisations initiated and funded the study, they had no role in the research process, data handling or interpretation, ensuring full scientific independence. All students attending Norwegian higher education institutions were eligible to participate in the SHoT. To date, five surveys assessing the health of students aged 18–35 in Norway have been conducted (2010, 2014, 2018, 2021 and 2022). Further information about the SHoT is available in previously published articles.^11^ The SHoT 2022 was conducted between 6 March and 19 April 2022.

In total, 169 572 students, encompassing all eligible full-time students enrolled in Norwegian higher education, received invitations to participate in the survey. Invitations were distributed via email and SMS, with two additional email reminders and one SMS reminder. Awareness was also raised through social media campaigns, outreach efforts by educational institutions, posters and digital advertisements on campuses. Of the invited students, 59 536 completed the survey, yielding a response rate of 35.1%. To address potential non-response bias, basic demographic information (age and gender) of respondents and non-respondents was compared. Non-respondents were slightly younger, but no significant gender differences were observed.

### Instruments

#### Hypochondriacal disorder

Self-reported hypochondriacal disorder was assessed using a comprehensive survey instrument listing mental and somatic health conditions relevant to this age group. Participants were asked if they had experienced any illnesses or disorders in the past 12 months. Selecting ‘mental disorders’ led to a secondary list of diagnostic categories, including ‘anxiety disorders’, where ‘hypochondriacal disorder’ was a selectable option.

The survey structure, including mental and somatic health categories and subcategories, is detailed in the supplementary file available at https://doi.org/10.1192/bjo.2025.68. This approach was adapted from validated methodology in the HUNT study.^12^ Participants’ self-reports did not require medical verification or diagnostic confirmation. For analysis, students were categorised into four groups: (a) hypochondriacal disorder only, (b) depression only, (c) comorbid hypochondriacal disorder and depression and (d) a control group reporting neither condition, although they may have had another health condition. This categorisation allowed for comparisons that distinguished unique and combined effects of hypochondriacal disorder and depression.

#### Sociodemographic information

Participants reported their age, gender and relationship status (categorised as single or married/partner/boyfriend/girlfriend). Gender responses included three categories: ‘woman’, ‘man’ and ‘other’. Ethnicity was classified as Norwegian if the participant or both parents were born in Norway.

#### Somatic symptom burden

Somatic symptom burden was measured using the Somatic Symptom Scale-8 (SSS-8), a validated self-report instrument based on the established Patient Health Questionnaire-15 (PHQ-15).^13^ In this study, the internal consistency of the SSS-8 was good (Cronbach’s *α* = 0.83). The scores were evaluated both as a continuous measure and as a binary variable, using a threshold of >11 to indicate a high or very high symptom burden. The term ‘somatic symptom burden’ was selected intentionally to clearly distinguish it from organic medical conditions. The SSS-8 assesses a range of common somatic symptoms, including stomach or bowel problems, chest pain or shortness of breath and feeling tired or having low energy. Participants rated how much they were bothered by these symptoms in the past 7 days on a five-point scale, ranging from ‘not at all’ to ‘very much’.

#### Mental distress

Mental health distress was evaluated using the Hopkins Symptoms Checklist (HSCL-25),^14^ a shorter version of the 90-item Symptom Checklist (SCL-90), designed for screening anxiety and depression symptoms. The HSCL-25 consists of 25 items rated on a Likert-type scale ranging from 1 (‘not at all’) to 4 (‘extremely’), referencing symptoms experienced over the previous 2 weeks. Previous findings from the SHoTs indicate that the HSCL-25 is unidimensional and is best understood as measure of ‘mental distress’ as it does not reliably differentiate between symptoms of anxiety and symptoms of depression.^15^ Validated cut-off scores (1.96 for males and 2.20 for females) were used as proxies for major depressive episodes (MDEs) or generalised anxiety disorder (GAD) in the past 30 days.^16^ The HSCL-25 includes items such as ‘feeling fearful’, ‘worrying too much about things’ and ‘feeling blue’, reflecting a range of internalising symptoms.

#### Sleep problems

Participants indicated the frequency per week of difficulties initiating sleep (DIS), difficulties maintaining sleep (DMS) and experiencing daytime tiredness or sleepiness. They also reported the duration of these sleep issues. Insomnia disorder was approximated based on DSM-5 criteria, defined as follows: (a) experiencing DIS or DMS at least 3 nights weekly, (b) experiencing daytime sleepiness or tiredness at least 3 days weekly and (c) persistence of these symptoms for at least 3 months. Additional information regarding the sleep inventory used in the SHoT has been detailed in earlier publications.^17^

#### Suicidal ideation, suicidal behaviour and self-harm

Suicidal ideation, suicidal behaviour and non-suicidal self-harm (NSSH) were assessed through four specific items. Three of these items were derived from the Adult Psychiatric Morbidity Survey (APMS),^18^ addressing lifetime history of suicidal thoughts, suicide attempts and NSSH behaviours. Participants responded ‘yes’ or ‘no’ to the following questions: ‘Have you ever seriously considered taking your own life without attempting it?’, ‘Have you ever attempted suicide by overdose or other means?’ and ‘Have you ever intentionally harmed yourself without suicidal intent?’ In addition, one item adapted from the Child and Adolescent Self-harm in Europe (CASE) study^19^ measured thoughts of NSSH: ‘Have you ever seriously thought about intentionally harming yourself without wanting to die, but never acted on these thoughts?’ Further details on assessing suicidality and NSSH in the SHoT are described elsewhere.^20^

#### Quality of life

Quality of life was measured using the Satisfaction With Life Scale (SWLS),^21^ a five-item questionnaire assessing participants’ overall cognitive evaluation of their life satisfaction, distinct from positive or negative emotional states. Respondents rated their agreement with each statement on a scale from 1 (strongly disagree) to 7 (strongly agree). The scale demonstrated strong internal consistency in the present study (Cronbach’s *α* = 0.87). SWLS scores were analysed both as a continuous measure and dichotomously, with scores of 19 or below indicating low life satisfaction. Example items from the SWLS include ‘In most ways my life is close to my ideal’, ‘The conditions of my life are excellent’ and ‘I am satisfied with my life’, which reflect a general sense of contentment and well-being.

#### Loneliness

Loneliness was evaluated using the Three-Item Loneliness Scale (T-ILS), an abbreviated form of the University of California, Los Angeles (UCLA) Loneliness Scale.^22^ Participants responded to three statements reflecting their experiences over the past year: (a) ‘How often do you feel that you lack companionship?’, (b) ‘How often do you feel left out?’ and (c) ‘How often do you feel isolated from others?’ Each item was rated on a five-point Likert scale ranging from ‘never’ to ‘very often’. The T-ILS demonstrated good internal consistency (Cronbach’s *α* = 0.84) and has previously shown strong reliability and validity. Scores were analysed both continuously and as a binary measure, categorising participants who reported ‘often’ or ‘very often’ on all three items as experiencing high loneliness, with all others classified as having low loneliness. Additional details regarding loneliness assessment in the SHoT have been previously published.^23^

### Statistical analyses

We conducted all analyses using IBM SPSS Statistics 29 for Windows (SPSS Inc., Chicago, IL, USA). Group differences in demographic variables were assessed using chi-square tests and independent sample *t*-tests on unweighted data. All remaining analyses exploring associations between hypochondriacal disorder and mental and somatic health outcomes applied weighting by gender to adjust for differences in gender distribution between the study sample and the broader student population. Weighting for socioeconomic status was not performed, as relevant population-level data were unavailable.

To allow comparisons across different measures, continuous scores for mental and somatic health were adjusted for age and gender, and then standardised into *t*-scores. Between-group differences were quantified using Cohen’s *d*, calculated from pooled standard deviations. These effect sizes were interpreted following Cohen’s guidelines: 0.20 indicates a small effect, 0.50 represents a moderate effect and above 0.80 suggests a large effect. For dichotomous outcomes, Poisson regression models with a log link function and robust standard errors were used, also adjusting for age and gender. Results from these models are presented as risk ratios with corresponding 95% confidence intervals, obtained by exponentiating regression coefficients. To ensure appropriateness of parametric analyses, the normality of continuous variables (HSCL-25, SWLS, SSS-8 and T-ILS) was assessed via skewness and kurtosis; all values fell within acceptable limits (±2).^24^ Overall, there were minimal missing data, so missing values were managed through listwise deletion. Given that the SHoT was designed with multiple research goals and was not specifically intended for examining hypochondriacal disorder, formal power analyses were not conducted beforehand to confirm adequate sample sizes for detecting group differences on the studied variables.

### Ethics

This report adheres to the Strengthening the Reporting of Observational Studies in Epidemiology (STROBE) reporting guidelines. The 2022 SHoT received ethical approval from the Regional Committee for Medical and Health Research Ethics in Western Norway (approval no. 326437). Participants provided electronic informed consent following a detailed explanation of the study procedures.

## Results

### Sample characteristics

In total, 457 participants reported having hypochondriacal disorder, corresponding to 0.86% of the full SHoT 2022 sample (*N* = 53 354). Of these, 203 participants (48, 0.38% of the full sample) had hypochondriacal disorder only, while 236 participants (52, 0.44% of the full sample) had comorbid hypochondriacal disorder and depression. As shown in Table 1, the proportion of females was significantly higher in all groups with hypochondriacal disorder and/or depression, with the highest proportion in the comorbid group (72.9%). Participants with hypochondriacal disorder (both groups) were also slightly older on average compared to those without these conditions. Regarding marital status, the proportion of single individuals was slightly lower in the hypochondriacal disorder-only group, but no significant differences were found for the other groups. For ethnicity, there were significant differences, with the lowest proportion of individuals with an immigrant background in the hypochondriacal disorder-only group (5.0%) and the highest in the comorbid group (16.9%).

**Table 1:** Descriptive characteristics of students by hypochondriacal disorder and depression status

Characteristics	(A) Control group (*n* = 46 381)	(B) Hypochondriacal disorder only (*n* = 203)	(C) Depression only (*n* = 6546)	(D) Comorbid hypochondriacal disorder and depression (*n* = 236)	Group differences^a^
Age, mean (s.d.)	23.94 (3.22)	24.27 (3.28)	24.51 (3.41)	24.78 (3.42)	A, B < C, D
Gender					
Females, n (%)	26 264 (56.6)	141 (69.5)	4579 (70.0)	172 (72.9)	A < B, C, D
Marital status					
Single, *n* (%)	23 641 (51.0)	100 (49.5)	3618 (55.3)	116 (49.2)	B < A
Ethnicity					
Self and/or parents born abroad, *n* (%)	4748 (10.2)	10 (5.0)	742 (11.3)	40 (16.9)	B < A < C < D

a. Group differences by hypochondria levels were tested with univariate analysis of variance (UNIANOVA), adjusting for age. Post hoc comparisons used least significant difference adjustment at *α* = 0.05.

### Somatic and mental health problems

**Fig. 1:**
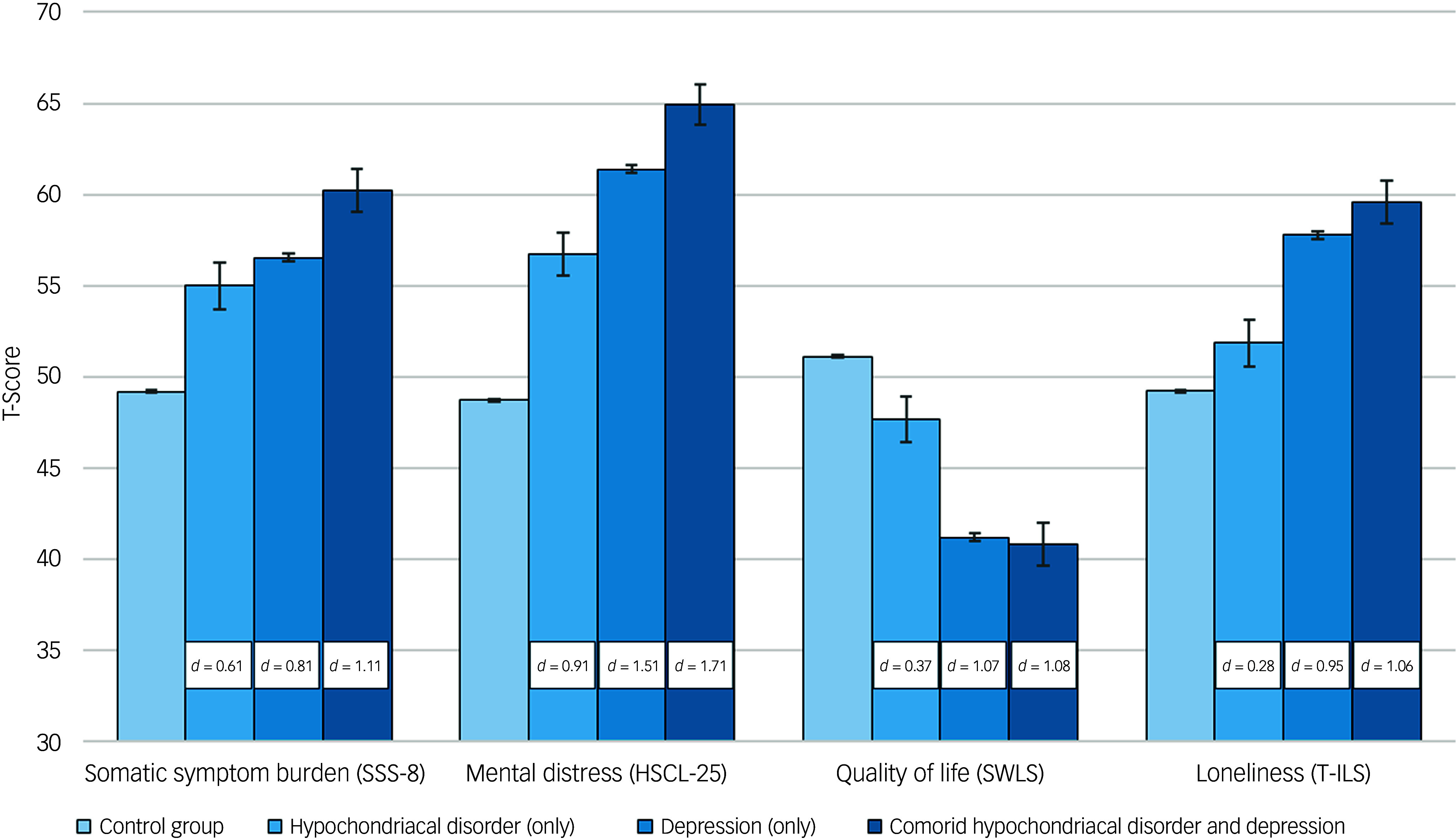
Somatic and mental health characteristics by hypochondriacal disorder and depression status. Scores are presented as standardised *t*-scores, adjusted for age and gender. The white boxes indicate Cohen’s *d* values, representing effect sizes relative to the control group for each measure. SSS-8, Somatic Symptom Scale-8; HSCL-25, Hopkins Symptoms Checklist; SWLS, Satisfaction With Life Scale; T-ILS, Three-Item Loneliness Scale.

As shown in Fig. 1, participants with hypochondriacal disorder reported higher levels of distress and impairment compared to the control group across all measures. Those with depression only had even higher scores, while participants with comorbid hypochondriacal disorder and depression reporting the highest symptom burden. For mental distress (HSCL-25), effect sizes relative to the control group ranged from *d* = 0.91 (hypochondriacal disorder only) to *d* = 1.71 (comorbid group), reflecting increasing symptom severity. A similar pattern was observed for somatic symptom burden (SSS-8 total), where effect sizes ranged from *d* = 0.61 (hypochondriacal disorder only) to *d* = 1.11 (comorbid group). For loneliness (T-ILS), scores were significantly higher in all affected groups compared to the control group, with the highest levels in the comorbid group (*d* = 1.06), followed by depression only (*d* = 0.95) and hypochondriacal disorder only (*d* = 0.28). Quality of life (SWLS) was lowest in the comorbid group, with an effect size of *d* = 1.08, followed by depression only (*d* = 1.07) and hypochondriacal disorder only (*d* = 0.37), indicating substantial reductions in life satisfaction across groups.

As detailed in Table 2, students with hypochondriacal disorder, depression or both had a significantly higher risk of poor mental and somatic health outcomes compared to the control group. Across the dichotomous measures, the comorbid group showed the highest burden, followed by the depression-only group, with the hypochondriacal disorder-only group reporting the lowest – but still elevated – risk. This pattern was consistent with the findings from the continuous outcome measures. The risk of high somatic symptom burden (SSS-8 > 11) was significantly elevated in all groups compared to the control group. The relative risk was 1.68 for students with hypochondriacal disorder, 1.81 for those with depression only and 2.24 for the comorbid group, where 73.8% met the high symptom burden threshold. Similarly, the risk of mental distress (HSCL-25 above cut-off) was significantly higher in all groups. The risk ratio was 2.56 for those with hypochondriacal disorder, 3.43 for depression only and 3.97 for the comorbid group, where 85.0% met the threshold. The risk of DSM-5 insomnia disorder was also elevated, with a risk ratio of 1.61 for hypochondriacal disorder, 2.07 for depression only and 2.29 for the comorbid group.

**Table 2:** Risk and prevalence of poor somatic and mental health in students with hypochondriacal disorder and depression

Health outcomes	Control group		Hypochondriacal disorder		Depression (only)		Comorbid hypochondriacal disorder and depression	
	Adj. risk ratio	(%)	Adj. risk ratio (95% CI)	(%)	Adj. risk ratio (95% CI)	(%)	Adj. risk ratio (95% CI)	(%)
Somatic symptoms (SSS-8 > 11)	1.00	(32.9)	1.68 (1.40–1.99)	(55.2)	1.81 (1.75–1.87)	(59.7)	2.24 (1.94–2.57)	(73.8)
Mental health distress (above HSCL-25 cut-off)	1.00	(21.4)	2.56 (2.12–3.06)	(54.9)	3.43 (3.31–3.55)	(73.5)	3.97 (3.45–4.53)	(85.0)
Insomnia disorder (DSM-5)	1.00	(29.0)	1.61 (1.32–1.94)	(47.0)	2.07 (2.00–2.14)	(60.0)	2.29 (1.96–2.65)	(67.0)
NSSH (lifetime)	1.00	(15.5)	1.94 (1.53–2.43)	(30.2)	2.98 (2.86–3.10)	(46.3)	3.08 (2.58–3.63)	(47.8)
NSSH thoughts (lifetime)	1.00	(17.7)	1.57 (1.21–1.99)	(27.8)	3.03 (2.91–3.15)	(53.6)	3.03 (2.56–3.56)	(53.6)
Suicide attempt (lifetime)	1.00	(2.9)	2.04 (1.12–3.36)	(5.8)	5.96 (5.51–6.45)	(17.0)	3.52 (2.34–5.04)	(10.0)
Suicide thoughts (lifetime)	1.00	(17.2)	1.40 (1.05–1.82)	(24.1)	3.58 (3.44–3.71)	(61.6)	3.21 (2.70–3.77)	(55.3)
Poor quality of life (SWLS < 19)	1.00	(25.6)	1.45 (1.15–1.80)	(37.2)	2.58 (2.49–2.68)	(66.1)	2.48 (2.11–2.90)	(63.5)
Loneliness (‘often’ or ‘very often’ on all 3 items)	1.00	(11.0)	1.51 (0.99–2.18)	(17.0)	4.24 (4.01–4.47)	(48.0)	5.16 (4.20–6.27)	(58.0)

Adj., adjusted; SSS-8, Somatic Symptom Scale-8 (scores >11 indicate high symptom burden); HSCL-25, Hopkins Symptom Checklist-25; DSM-5, *Diagnostic and Statistical Manual of Mental Disorders* (5th edn); NSSH, non-suicidal self-harm; SWLS, Satisfaction with Life Scale (scores <19 indicate low life satisfaction). All analyses are weighted for gender; risk ratios and prevalence estimates are adjusted for age and gender.

Participants with hypochondriacal disorder also had an increased risk of NSSH behaviours and suicidality. The risk of lifetime NSSH was 1.94 for hypochondriacal disorder, 2.98 for depression only and 3.08 for the comorbid group. A similar pattern was observed for NSSH thoughts (lifetime), with risk ratios of 1.57, 3.03 and 3.03, respectively. For lifetime suicide attempts, the risk ratio was 2.04 for hypochondriacal disorder, significantly lower than for depression only (5.96) or the comorbid group (3.52). The risk of lifetime suicidal thoughts was 1.40 for hypochondriacal disorder, 3.58 for depression only and 3.21 for the comorbid group. Regarding low quality of life (SWLS < 19), the risk ratio was 1.45 for hypochondriacal disorder, 2.58 for depression only and 2.48 for the comorbid group. Finally, loneliness (frequently endorsing all three loneliness indicators) was significantly elevated in all groups, with the highest risk ratio in the comorbid group (5.16).

## Discussion

In this national survey of Norwegian university and college students, 0.86% (457 participants) reported hypochondriacal disorder, with around half also experiencing comorbid depression. This overlap highlights the complexity of these conditions. Students with hypochondriacal disorder had significantly worse outcomes than controls, with the most severe impairments in those with comorbid depression. While the symptom burden of hypochondriacal disorder alone was comparable to – or slightly lower than – that of depression, comorbid students showed the highest level of impairments across most measures. These findings highlight the need for targeted interventions addressing hypochondriacal disorder and its common comorbidities.

Our findings reveal a clear association among hypochondriacal disorder, mental distress and somatic symptom burden. Students with hypochondriacal disorder experienced high levels of mental distress, with a large effect size (*d* = 0.91), although this was even more pronounced in those with depression (*d* = 1.29) and highest in students with both conditions (*d* = 1.71). Similarly, the relative risk of mental distress increased from 2.56 in those with hypochondriacal disorder alone to 3.97 in the comorbid group. A similar pattern emerged for somatic symptom burden. While hypochondriacal disorder alone was linked to a moderate effect (*d* = 0.61), this increased in students with depression (*d* = 0.87) and was highest in those with both conditions (*d* = 1.11). The relative risks followed the same trend, rising from 1.68 to 2.24 in comorbid students. These results suggest that while hypochondriacal disorder has a significant impact on both mental and somatic health, the presence of depression considerably amplifies this burden. The consistent increase in both effect sizes and relative risks across groups highlights the compounded effect of comorbidity, emphasising the need for integrated approaches to assessment and treatment.

There is a notable scarcity of research examining the association between hypochondriacal disorder and adverse outcomes, particularly in relation to both somatic and mental health. Existing studies have focused primarily on clinical settings, and little is known about the disorder’s impact in student populations. However, some studies have highlighted a high level of comorbidity between hypochondriacal disorder and other mental health conditions. For example, our findings align with prior research indicating that major depression and anxiety disorders frequently co-occur with hypochondriacal disorder.^25–27^ A German university study also found that 22.7% of students met the criteria for at least one psychological disorder, with hypochondriacal disorder present in 4.2% of students, significantly increasing the risk of comorbid disorders.^28^

Our study is among the few to examine the link between hypochondriacal disorder and suicidality in a large student sample. Students with hypochondriacal disorder faced increased risks of suicidal thoughts (risk ratio 1.40) and suicide attempts (risk ratio 2.04). They also had a higher risk of significant somatic symptom burden (SSS-8 > 11; risk ratio 1.68) and DSM-5 insomnia disorder (risk ratio 1.61). These risks were markedly higher among students with comorbid depression, with risk ratios of 3.58 for suicidal thoughts and 5.96 for suicide attempts. This indicates that while hypochondriacal disorder contributes to suicidality, depression remains a stronger predictor.

The overlap between hypochondriacal disorder and depression emphasises the importance of integrated mental health interventions. More than half of students with hypochondriacal disorder also reported depression, highlighting the importance of routine screenings for depression in individuals presenting with health anxiety. Managing hypochondriacal disorder is crucial, but addressing underlying depressive symptoms may yield greater overall improvements, as students with depression alone often face similar or greater impairments across multiple domains compared to those with hypochondriacal disorder alone. Given the strong link between hypochondriacal disorder and suicidal ideation, suicide risk assessments should also be incorporated into clinical evaluations. Health practitioners should adopt a multidisciplinary approach, combining psychological therapies, such as cognitive–behavioural therapy (CBT), with interventions targeting related mental health concerns. Several studies have demonstrated the efficacy of CBT for hypochondriacal disorder,^29,30^ with emerging evidence suggesting long-term maintenance of treatment gains.^31^ Given the substantial overlap between hypochondriacal disorder and depression, adapted CBT approaches that address both conditions simultaneously may be particularly beneficial. Addressing associated issues, such as insomnia and loneliness, is likely important, as these factors were significantly elevated across all affected groups. Campus health centres should provide resources for sleep management, social support and counselling services. Ultimately, improving student mental health requires comprehensive interventions that account for the full spectrum of psychiatric comorbidities rather than focusing on hypochondriacal disorder in isolation.

Several methodological considerations should be mentioned regarding our study. Key strengths include the large sample size and the use of validated instruments. This study provides new insights into the associations between hypochondriacal disorder and a wide range of somatic and mental health outcomes among higher education students in a post-COVID-19 pandemic national sample. However, the cross-sectional design prevents us from inferring causality or determining the direction of observed associations. Longitudinal research designs would better elucidate the nature and direction of these relationships. Another limitation is the modest response rate of 35.1%, with limited information on non-participants other than age and gender. Selective participation may have introduced bias in the strength of observed associations, as non-participants in health surveys typically report poorer general health,^32^ and individuals are more likely to participate in surveys they find personally relevant.^33^ In addition, the absence of weighting for socioeconomic status may have affected the study outcome. Furthermore, our reliance on self-report measures, rather than clinical diagnoses, introduces potential biases. Self-reported data are subject to misclassification and individual interpretation, which may lead to variability in how hypochondriacal disorder is reported. This underscores the need for future research to combine self-report measures with clinical evaluations to ensure more precise assessments. Future research should employ longitudinal designs to better understand the progression of hypochondriacal disorder, its potential causal relationships and the long-term impact of comorbid mental health conditions. In addition, research on treatment approaches, particularly CBT for health anxiety, is warranted to assess its effectiveness in student populations. Exploring how different CBT modalities – such as digital interventions or brief therapy models – can be adapted for university settings may provide valuable insights for improving access to care and treatment outcomes.

In conclusion, hypochondriacal disorder is associated with significant health burdens in university students, with large effect sizes for mental distress and moderate to large effect sizes for somatic symptoms and loneliness. Depression often results in similar or greater impairment, with comorbid students showing the worst outcomes. These findings highlight the need for comprehensive mental health strategies in educational settings, integrating screening, early intervention and evidence-based treatments for hypochondriacal disorder and its frequent comorbidities, particularly depression.

## Supporting information

Veddegjærde et al. supplementary material Veddegjærde et al. supplementary material

## Data Availability

Data sharing is restricted by Norwegian data protection regulations and General Data Protection Regulation (GDPR). However, researchers can request access to participant data by contacting the SHoT publication committee (borge.sivertsen@fhi.no). Data access requires approval from the Norwegian Regional Committee for Medical and Health Research Ethics (https://helseforskning.etikkom.no). The data-set is managed by the Norwegian Institute of Public Health (NIPH), and further information regarding data access procedures is available at https://www.fhi.no/en/more/access-to-data. The analytic code supporting the reported findings is available upon request from the corresponding author (B.S.).

## Supplementary material

The supplementary material is available online at https://doi.org/10.1192/bjo.2025.68
