# Supplementary material for: Mental and somatic health burdens of hypochondriacal disorder in higher education: national study among Norwegian students
Source: BJPsych Open. 2025 May 15;11(3):e108. doi: 10.1192/bjo.2025.68 (PMC12089818; doi:10.1192/bjo.2025.68)
Supplement: Veddegjærde et al. supplementary material [file S2056472425000687sup001.docx]

*This file outlines the survey structure, including mental and somatic health categories and subcategories.*

Somatic and Mental Health Conditions
*Have you had any of the following diseases or conditions in the past 12 months?*

- Allergy and intolerances
  - Celiac disease
  - Year-round allergies (e.g., house dust mites, animals)
  - Contact allergy (e.g., perfume, nickel)
  - Food allergies (e.g., milk, eggs, shellfish, nuts)
  - Food intolerance (non-allergic)
  - Seasonal allergies (pollen)
- Asthma
- Cerebral Palsy
  - Unilateral
  - Bilateral
- Diabetes
  - Type 1 diabetes
  - Type 2 diabetes
  - Gestational diabetes
  - LADA
  - MODY
- Eczema
  - Atopic eczema
  - Contact eczema (hand eczema)
  - Other (specified)
- Epilepsy
  - Generalized seizures
  - Focal seizures
  - Unclassified/unknown seizures
- Fibromyalgia
- Heart Disease
  - Congenital heart defect (operated/not operated)
  - Cardiomyopathy
  - Arrhythmia (e.g., SVT, atrial fibrillation)
  - Palpitations ("extra beats")
  - Hereditary heart disease (genetic defect)
  - Coronary artery disease
  - Other (specified)
- Hearing Problems
  - Deafness
  - Reduced hearing
  - Tinnitus
- Irritable Bowel Syndrome (IBS)
- Sexually Transmitted Diseases
  - Chlamydia
  - Genital herpes
  - Genital warts
  - Lymphogranuloma venereum (LGV)
  - HIV infection
  - Mycoplasma
  - Gonorrhea
  - Syphilis
  - Hepatitis C
  - Hepatitis B
  - Other (specified)
- Cancer
  - Breast cancer
  - Melanoma
  - Brain tumor
  - Skin cancer
  - Leukemia
  - Cervical cancer
  - Lymphoma
  - Bone or muscle cancer
  - Testicular cancer
  - Other (specified)
- Chronic Fatigue Syndrome/ME
- Mental Disorders
  - ADHD/ADD
    - ADHD (Attention Deficit Hyperactivity Disorder)
    - ADD (Attention Deficit Disorder)
  - Anxiety Disorders
    - Generalized anxiety disorder
    - Agoraphobia
    - Panic disorder
    - Specific phobias
    - **Hypochondriacal disorder**
    - Social anxiety
  - Autism Spectrum Disorders
    - Autism
    - Asperger syndrome
  - Bipolar Disorder
  - Depression
  - Dissociative Disorder
  - Personality Disorder
  - Psychosis
  - PTSD (Post-Traumatic Stress Disorder)
  - Schizophrenia
  - Eating Disorders
    - Bulimia nervosa
    - Anorexia nervosa
    - Binge-eating disorder
  - Obsessive-Compulsive Disorder (OCD)
  - Tourette Syndrome
  - Other (specified)
- Rheumatoid Arthritis
- Migraine
  - Migraine with aura
  - Migraine without aura
- Multiple Sclerosis (MS)
- Visual Problems
  - Blindness
  - Visual impairment
- Other disease/condition (specified)
